# A luminescent reporter assay to quantify SORL1 ectodomain shedding and retromer-dependent endosome recycling activity

**DOI:** 10.1016/j.jbc.2026.111136

**Published:** 2026-01-08

**Authors:** Elnaz Fazeli, Asad Jan, Ann-Kathrin Huber, Anne Mette G. Jensen, Elham Fazeli, Joel Klein, Kalpana Merchant, Olav M. Andersen

**Affiliations:** 1 Department of Biomedicine, Aarhus University, Aarhus C, Denmark; 2 Department of Bioengineering, The Weihenstephan-Triesdorf University of Applied Science, Freising, Germany; 3 Retromer Therapeutics, New York, New York, USA; 4 Northwestern University Feinberg School of Medicine, Chicago, Illinois, USA

**Keywords:** ectodomain shedding, *Gaussia* luciferase, luminescent reporter, retromer, *SORL1*

## Abstract

Growing evidence suggests that defects in endosomal recycling are a causal mechanism for Alzheimer’s disease (AD). Sortilin-like receptor 1 (SORL1) is an endosomal sorting receptor that acts together with the retromer complex to facilitate shuttling of cargo from endosomes back to the *trans*-Golgi network or to the cell surface. Accumulating data indicate that SORL1 dysfunction contributes to AD pathogenesis. SORL1 is trafficked from the endosome to the cell surface in a retromer-dependent process, where it undergoes enzymatic cleavage, resulting in shedding of the SORL1 ectodomain (also known as soluble SORL1). We capitalized on this physiological process to develop and validate a cell-based luminescent reporter assay incorporating enhanced *Gaussia* luciferase (eGLuc) to quantify soluble SORL1 in the conditioned media as a marker of endosomal recycling function. The shedding of eGLuc–SORL1 provided a reliable luminescent readout correlating with cellular SORL1 expression under conditions of stable and transient transfection in mammalian cell cultures. Using this system, we demonstrated a robust dependence of SORL1 shedding on retromer levels. Pharmacological treatments and manipulations that either inhibited or enhanced retromer activity produced corresponding changes in eGLuc–SORL1 shedding. Furthermore, the assay demonstrated a reduction in SORL1 shedding in cells expressing pathogenic variants associated with AD, supporting its application in evaluating variant pathogenicity. Given its simplicity and cost-effectiveness, this assay is well suited for high-throughput screening of small-molecule drug candidates that modulate SORL1 trafficking and endosomal recycling. In a broader context, it provides a valuable tool for investigating the biological significance of AD-associated *SORL1* variants.

Alzheimer’s disease (AD) is a progressive neurodegenerative disorder and the leading cause of dementia worldwide (1). While the identity of specific factor(s) triggering neurodegenerative processes in AD remains a matter of active debate, emerging evidence points to defective endosomal recycling as a causal mechanism (2). A key component of the endosomal recycling pathway is the retromer complex, often described as “the master conductor” of endosomal trafficking (3). Retromer activity directs cargo from endosomes to the *trans*-Golgi network or, very critically for neuronal fitness, back to the cell surface (3, 4). Central to this mechanism is the sortilin-like receptor 1 (SORL1), an endosomal sorting protein that acts as a receptor and binding partner for retromer, shuttling cargo such as amyloid precursor protein, α-amino-3-hydroxy-5-methyl-4-isoxazolepropionic acid receptors, and neurotrophin receptors (4, 5, 6). Previous studies detailed how SORL1 interacts with the retromer complex, contributing to a scaffold that stabilizes the tubular membrane structure, which is essential for transporting cargo out of endosomes (7, 8).

Several lines of evidence indicate that defective SORL1 activity contributes to AD development (9). SORL1 deficiency in cell and animal models impairs endosomal recycling and triggers key AD hallmarks, such as amyloid and tau pathologies, synaptic dysfunction, and enlarged endosomes (4, 6, 10, 11). In addition to SORL1's contribution to the hallmark cellular processes of AD, genetic studies have shown how *SORL1* variants are associated with high AD risk (12, 13, 14, 15, 16). Nonsense or frameshift variants that introduce premature stop codons downstream in the *SORL1* gene, resulting in loss of protein function, occur almost exclusively in AD patients (16). Moreover, some *SORL1* missense variants have been reported to cosegregate with AD with an autosomal dominant inheritance pattern (17, 18). To date, over 500 *SORL1* coding variants have been identified in AD patients, with their impact on protein function ranging from minimal to highly deleterious. Understanding the significance of AD-associated *SORL1* variants, especially their functional consequences on cellular SORL1 activity, is being investigated (19).

SORL1 is synthesized in the endoplasmic reticulum (ER) and transferred to the Golgi, from where it can be directed to the endosome and subsequently to the cell surface. SORL1 N-terminal propeptide is proteolytically cleaved during this pathway (20, 21). We have previously shown how trafficking of SORL1 in the endosome recycling pathway is required for its maturation process, requiring a number of complex-type *N*-glycosylations and subsequent shedding from the cell surface after proteolytic cleavage by the tumor necrosis factor alpha–converting enzyme (TACE). This processing step leads to shedding of the large soluble ectodomain (sSORL1) (22), suggesting that sSORL1 can serve as a biological readout for endosomal recycling activity. Supporting this idea, recent studies suggest that certain AD-associated missense *SORL1* variants exhibit altered maturation and propensity for retention within the ER, with consequent reduction in the delivery of SORL1 to the endosome. As a result, less mature receptor traffics to the cell surface, leading to reduced production of sSORL1 (7, 18, 19, 23). Thus, a cellular system that can accurately and quantitatively monitor sSORL1 offers a method to study the efficiency of the retromer-dependent endosomal recycling pathway.

The *Gaussia* luciferase (GLuc) is an enzyme originally isolated from the marine *Gaussia princeps* (24) and has been used as a reporter for a variety of biological processes because of its high sensitivity (25). GLuc is a naturally secreted protein, and it has been shown to be over 20,000-fold more sensitive in monitoring the secretory pathway in mammalian cells compared with the most commonly used secreted reporter, secreted embryonic alkaline phosphatase (26). Importantly, optimization by mutating two oxidation-prone methionines (M43 and M110) has enabled the engineering of an enhanced version of the *Gaussia* luciferase (eGLuc) enzyme with improved extracellular stability (27, 28). Here, we describe a luminescent reporter assay based on a cellular system expressing eGLuc fused to the N terminus of SORL1 for monitoring sSORL1 production in samples from the extracellular milieu. We demonstrated that this system accurately quantifies sSORL1 with high efficiency and specificity.

## Results

### The eGLuc–SORL1 reporter construct is a robust indicator of SORL1 shedding

**Figure 1:**
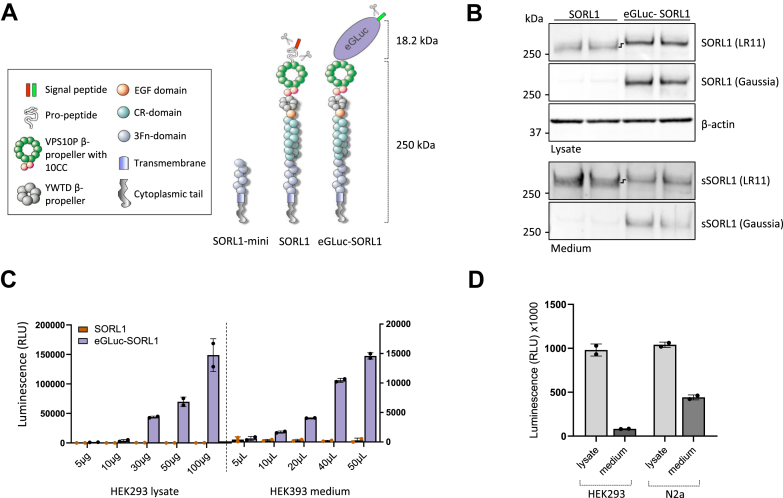
**Generation and validation of the eGLuc–SORL1 reporter construct.***A*, schematic representation of the structural elements of SORL1 minireceptor (SORL1-mini), SORL1, and eGLuc–SORL1 reporter (with sizes in kilodaltons indicated to the *right*). Scissors indicate proteolytic cleavage sites that remove the signal peptide and propeptide. *B*, representative immunoblots of cell lysates and conditioned medium from N2a cells transfected with expression constructs for SORL1 (*i.e.*, original vector without fusion to GLuc) and eGLuc–SORL1, measured after 24 h of shedding, detection antibodies as indicated. *C*, eGLuc activity measured from increasing amounts of cell lysates and conditioned medium from HEK293 cells transfected with SORL1 or eGLuc–SORL1. Luminescence signal is measured in medium allowed to accumulate shed fragments for 24 h and is expressed in relative light units (RLUs). n = 1 independent experiment, with two technical replicates in each. Error bars indicate mean ± SD. *D*, comparison of eGLuc activity in cell lysates and conditioned medium from HEK293 and N2a cells transfected with eGLuc–SORL1. Luminescence was measured after 24 h of shedding and is expressed in RLUs. n = 1 independent experiment with two technical replicates. Error bars indicate mean ± SD. eGLuc, enhanced *Gaussia* luciferase; HEK293, human embryonic kidney 293 cell line; SORL1, sortilin-like receptor 1; sSORL1, soluble SORL1.

Here, we develop and validate a quantitative cell-based assay for measuring the shedding of SORL1, which relies on the enzymatic activity of eGLuc. For this purpose, we engineered a fusion DNA construct of SORL1 fused to the eGLuc in-frame to the N-terminal end of the receptor, substituting the native signal peptides and propeptides with the *Igκ leader* sequence and the enzymatic reporter in front of the VPS10p domain beginning at amino acid 82 (29), since SORL1 does not require the propeptide for normal processing and transport (30). In addition, we included a 5-residue linker (DYKKD) following the eGLuc moiety, allowing the VPS10p domain to fold efficiently in agreement with a previous report showing that the SORL1 propeptide is not required for the folding of the SORL1 VPS10p domain (31) (Fig. 1*A*).

Then, we transfected human embryonic kidney 293 (HEK293) and N2a cells with eGLuc–SORL1 reporter construct (1.5 and 3.0 μg, respectively, in 6-well format; see the *Experimental procedures* section) using a standard protocol with the nonliposomal FuGene reagent. We evaluated the protein expression and shedding of the reporter (eGLuc–SORL1) in comparison to the nontagged, full-length receptor (SORL1) by immunoblotting of cell lysates and conditioned media, respectively, in N2a cells (Fig. 1*B*). Detection with a SORL1-directed antibody (LR11) indicated efficient expression of the reporter, in the form of a product that migrated slower than the native SORL1 because of the extra size of the eGLuc tag (∼18 kDa). Detection with an antibody specific for the Gaussia part confirmed the expression of the full-length fusion product (cell lysates) as well as the shed fusion fragment (conditioned media).

In parallel, we quantified the assay performance by measuring eGLuc activity (luminescence signal) in the cell lysates and conditioned media from HEK293 cells transfected with (1.5 μg/6-well) SORL1 or eGLuc–SORL1. For this purpose, we sampled increasing amounts of the cell lysates or volumes of the conditioned media, which exhibited correspondingly higher luminescence signal by the reporter construct, whereas no signal was detectable in samples from cells transfected with the native WT SORL1 (Fig. 1*C*).

We then compared the eGLuc signal in lysate and medium between HEK293 and N2a cells. Consistent with our previous findings for the untagged receptor (19), we observed a higher shedding of the eGLuc–SORL1 reporter in N2a cells compared with the HEK293 cell line (Fig. 1*D*).

### eGLuc–SORL1 exhibits intracellular localization to endosomes

**Figure 2:**
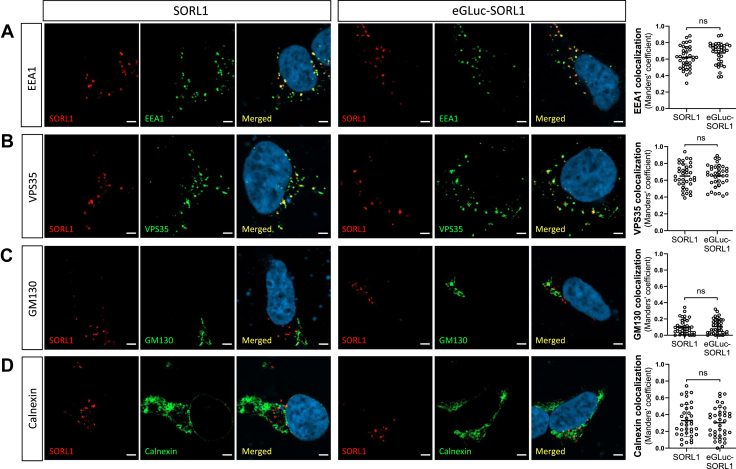
**Cellular localization of eGLuc–SORL1 and SORL1.** Representative images showing immunofluorescence of HEK293 cells transfected with SORL1 or eGLuc–SORL1 and immunostained with an antibody for SORL1 (*red*) and a panel of different marker antibodies against (*A*) EEA1, (*B*) VPS35, (*C*) GM130, or (*D*) calnexin (*green*) as well as a nuclear stain with DAPI (*blue*). The scale bars represent 5 μm. Graphs represent quantification of Manders’ colocalization coefficients between SORL1/eGLuc–SORL1 and EEA1, VPS35, GM130, and calnexin as indicated. Each data point represents one cell (one image per cell); 30 to 40 individual cells were analyzed per condition from n = 2 independent experiments. Error bars indicate mean ± SD. Data were analyzed using a parametric two-tailed unpaired *t* test (ns, not significant). DAPI, 4′,6-diamidino-2-phenylindole; EEA1, early endosome antigen 1; eGLuc, enhanced *Gaussia* luciferase; HEK293, human embryonic kidney 293 cell line; SORL1, sortilin-like receptor 1.

We have previously reported that intracellular SORL1 predominantly localizes to early endosome antigen 1 (EEA1)–positive endosomes and retromer-coated tubules of the endosome in transfected HEK293 cells (7, 18). In comparison, a minor fraction of the receptor is observed in the early compartments of the secretory pathway, including the ER, as identified by staining for calnexin (18, 19). Therefore, we investigated the intracellular localization of eGLuc–SORL1, using codetection with markers of EEA1, retromer (VPS35), *cis*-Golgi (GM130), and ER (calnexin) in HEK293 cells transfected with either SORL1 or eGLuc–SORL1 construct (Fig. 2). Immunofluorescence analyses by confocal imaging and quantification of the colocalization coefficients showed that eGLuc–SORL1 and WT SORL1 had identical cellular localization, at least when assayed for this panel of cellular markers. The Manders’ colocalization values were as follows: EEA1 (Fig. 2*A*; SORL1: 0.62 ± 0.14 *versus* eGLuc–SORL1: 0.67 ± 0.13, ns), VPS35 (Fig. 2*B*; SORL1: 0.65 ± 0.14 *versus* eGLuc–SORL1: 0.65 ± 0.13, ns), GM130 (Fig. 2*C*; SORL1: 0.10 ± 0.09 *versus* eGLuc–SORL1: 0.11 ± 0.09, ns), and calnexin (Fig. 2*D*; SORL1: 0.33 ± 0.19 *versus* eGLuc–SORL1: 0.30 ± 0.18, ns). These data are promising indicators that the intracellular distribution of eGLuc–SORL1 reporter is similar to WT native SORL1, and the addition of eGLuc in the N terminus has minimal effects on the cellular trafficking, at least within the applied conditions in these experiments (Fig. 2).

We aimed to further validate and optimize the delivery of eGLuc–SORL1 and assay conditions to ensure robust detection of the shedding. In brief, we compared different transfection methods, assessed SH-SY5Y cells for their ability to express and shed eGLuc–SORL1 relative to HEK293T, and tested different time points for luminescence readout. These experiments confirmed that Magnetofectamine transfection reagent exhibited higher transfection efficiency compared with FuGene, which was reflected by higher eGLuc–SORL1 expression and shedding (Fig. S2*A*). In these experiments, we also observed that SH-SY5Y cells exhibited moderately higher shedding than HEK293T cells (Fig. S2*B*), and reliable luminescence signals were detected as early as 2 to 3 h after media change (Fig. S2*C*).

### Biological validation of eGLuc–SORL1

We and others have previously shown that the shedding of sSORL1 in cultured cells can be pharmacologically modulated by small molecules (22, 32). For instance, phorbol 12-myristate 13-acetate (PMA), a chemical compound that leads to increased TACE activity at the cell surface, increased SORL1 shedding, which potentially emanates from higher proteolytic cleavage capacity at the cell surface (32). Also, treatment of cells overexpressing SORL1 with Dynasore (Dyn), a pharmacological inhibitor of dynamin-dependent endocytosis (33), results in decreased shedding of the native SORL1 protein; albeit, the mechanism remains to be clarified (22).

**Figure 3:**
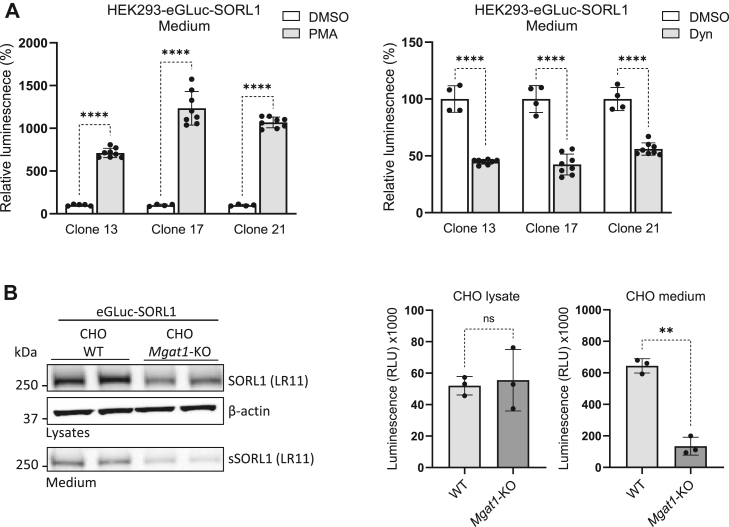
**Biological validation of eGLuc–SORL1 using pharmacological and genetic approaches.***A*, HEK293 cells stably expressing eGLuc–SORL1 (HEK293–eGLuc–SORL1) were treated with either 50 μM Dynasore (Dyn) or 160 nM phorbol 12-myristate 13-acetate (PMA) for 24 h and 2 h, respectively, before the medium was harvested and the luminescence signal was quantified. The treatment has been done on three independent clones of HEK293 cells with stable expression of eGLuc–SORL1 (clones 13, 17, and 21). Error bars indicate mean ± SD. Data were analyzed using a parametric two-tailed unpaired *t* test (∗∗∗∗*p* ≤ 0.0001). *B*, immunoblots of cell lysates and conditioned medium from CHO cells (WT or with KO of *Mgat1*) expressing eGLuc–SORL1 following transfection with Magnetofectamine reagents, detection antibodies as indicated. eGLuc activity measured in the cell lysate and conditioned media of CHO cells (WT or with KO of *Mgat1* (45)) expressing eGLuc–SORL1 following transfection with Magnetofectamine reagent. Signal measured after 24 h of shedding. n = 3 independent experiments with two technical replicates in each. Data were analyzed using a parametric two-tailed paired *t* test. (∗∗*p* ≤ 0.01; ns, not significant). Error bars indicate mean ± SD. CHO, Chinese hamster ovary; eGLuc, enhanced *Gaussia* luciferase; HEK293, human embryonic kidney 293 cell line; SORL1, sortilin-like receptor 1.

Here, we wanted to investigate if the performance of the eGLuc–SORL1 reporter assay can be pharmacologically modulated as a function of SORL1 shedding. For this purpose, we generated a number of HEK293 cell lines with stable expression of eGLuc–SORL1 (HEK293–eGLuc–SORL1). We then quantified the effect of Dyn or PMA treatments on eGLuc activity in the conditioned media. In brief, three distinct HEK293–eGLuc–SORL1 clones were selected based on Western blotting and immunostaining, which confirmed that they were monoclonal. Furthermore, each clone had a different expression level of the reporter. The clones were subsequently treated with PMA (160 nM), Dyn (50 μM), or dimethyl sulfoxide (DMSO) control, followed by measurements of luminescence signal. For each of the three clones, we observed a significant (∼50%) reduction in eGLuc–SORL1 shedding in cells treated with Dyn compared with DMSO. In contrast, a 7- to 12-fold increase in eGLuc–SORL1 shedding was detected in cells treated with PMA compared with DMSO. Together, these results are positive indicators that the eGLuc reporter responds similarly to these pharmacological interventions as the native SORL1, as previously reported (22) (Fig. 3*A*).

To also validate that *N*-glycosylation of eGLuc–SORL1 is required for its maturation and cell surface transport, similar to the native SORL1 (22), we expressed eGLuc–SORL1 in a glycoengineered Chinese hamster ovary (CHO) cell line with KO of the *Mgat1* gene, which encodes the key enzyme responsible for initiating complex-type *N*-glycosylation (34). CHO–WT cells and CHO-*Mgat1*-KO cells were separately transfected with the eGLuc–SORL1 construct, and shedding efficiency was analyzed by measuring the luciferase signal in the cell lysates and conditioned media. These studies indicated that although the expression of eGLuc–SORL1 was nearly identical between the two cell lines, extracellular shedding of eGLuc–SORL1 was considerably reduced in CHO-*Mgat1*-KO cells (Fig. 3*B*). This was also corroborated by Western immunoblotting analyses, which reflected the expression of the full-length eGLuc–SORL1 fusion protein in cell lysates and conditioned media, and confirmed the decreased shedding in the absence of cellular Mgat1 activity (Fig. 3*B*).

### Impaired retromer interaction/activity decreases shedding of eGLuc–SORL1

Previous studies indicate that SORL1 interacts with the retromer complex through the FANSHY motif located in the cytoplasmic tail of the receptor (5). In this context, we have previously reported that targeted mutagenesis with substitutions of the amino acids from the FANSHY motif with alanine residues (FANSHY>6A) disrupts the SORL1–retromer interaction and reduces ectodomain shedding in conditioned media (5, 22).

**Figure 4:**
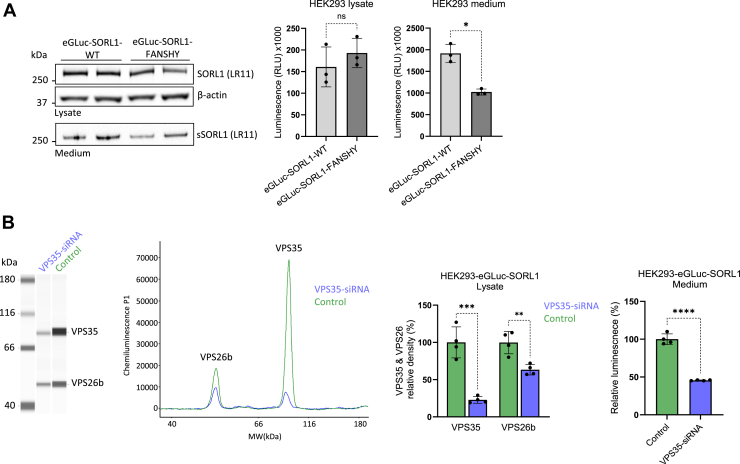
**eGLuc–SORL1 shedding depends on functional retromer interaction/activity.***A*, immunoblots of cell lysates and conditioned media from HEK293 cells expressing eGLuc–SORL1–WT or eGLuc–SORL1–FANSHY following transfection with Magnetofectamine reagents. Detection antibodies as indicated. eGLuc activity measured in the cell lysate and conditioned media of HEK293 cells expressing eGLuc–SORL1–WT or eGLuc–SORL1–FANSHY following transfection with Magnetofectamine reagents, measured after 24 h of shedding. n = 3 independent experiments with two technical replicates in each. Data were analyzed using a parametric two-tailed paired *t* test (∗*p* ≤ 0.05; ns, not significant). *B*, representative capillary Western lane view and graph readout of VPS35 and VPS26b expression levels from lysate of HEK293 cells stably expressing eGLuc–SORL1 (HEK293–eGLuc–SORL1) treated with either VPS35-siRNA or nontargeting siRNA (scrambled). Corresponding densitometry analyses are also shown; eGLuc activity measured in the conditioned media of HEK293 cells stably expressing eGLuc–SORL1 treated with either VPS35-siRNA or scrambled siRNA. n = 1 independent experiments with four technical replicates in each. Error bars indicate mean ± SD. Data were analyzed using a parametric two-tailed unpaired *t* test and shown as relative to the scrambled control (∗∗*p* ≤ 0.01; ∗∗∗*p* ≤ 0.001; and ∗∗∗∗*p* ≤ 0.0001). eGLuc, enhanced *Gaussia* luciferase; HEK293, human embryonic kidney 293 cell line; SORL1, sortilin-like receptor 1; sSORL1, soluble SORL1.

Therefore, we applied this approach for investigating the cellular expression and shedding of eGLuc–SORL1 in cultured HEK293T cells following transfections with either eGLuc–SORL1–WT or eGLuc–SORL1–FANSHY plasmid constructs. Western blot analysis of lysates and medium from the transfected cells showed similar expression levels of the two reporter constructs in cell lysates but indicated lower shedding of the eGLuc–SORL1–FANSHY compared with the native receptor. Using the same samples for measuring the luminescence signal showed a significant ∼50% reduction in the shedding of eGLuc–SORL1–FANSHY compared with eGLuc–SORL1–WT, supporting that the reporter assay can be used for detecting endosomal and retromer-dependent trafficking of SORL1 (Fig. 4*A*).

In order to consolidate that SORL1 shedding depends on retromer activity and to confirm that the eGLuc–SORL1 reporter responds similarly, we transiently knocked down VPS35 (a core subunit of retromer) in HEK293–eGLuc–SORL1 cells (clone 17) by siRNAs. Using an automated capillary-based Western system (Jess; ProteinSimple), we measured the knockdown efficiency in HEK293–eGLuc–SORL1 cells by comparing the expression levels of VPS35 and VPS26 (a subunit of retromer that is known to be coregulated with VPS35 (4)) *versus* nontargeting siRNA-treated cells. The expression of VPS35 protein in the cells transfected with the VPS35-siRNA was less than 25% compared with the control cells (Fig. 4*B*), along with ∼40% reduction in the expression of VPS26 (Fig. 4*B*). After retromer knockdown was confirmed, conditioned medium from these cells treated with either VPS35- or nontargeting siRNA was collected, and luminescence signal was measured. Here, we could quantify a notable 50% reduction in eGLuc–SORL1 shedding in the media of cells treated with VPS35-siRNA compared with control siRNA. This experiment was independently repeated in two additional HEK293–eGLuc–SORL1 clones (clones 13 and 21), and the reduction in shedding was consistently observed in both clones (Fig. S3). Collectively, these data support the role of the retromer complex in the recycling and subsequent shedding of eGLuc–SORL1 (Figs. 4*B* and S3).

### Boosting retromer activity/recycling pathway increases eGLuc–SORL1 shedding

Having established how retromer activity can be quantified using the eGLuc–SORL1 shedding assay, we wanted to use our assay to assess the effect of recently suggested methods of increasing retromer and endosome recycling activities.

**Figure 5:**
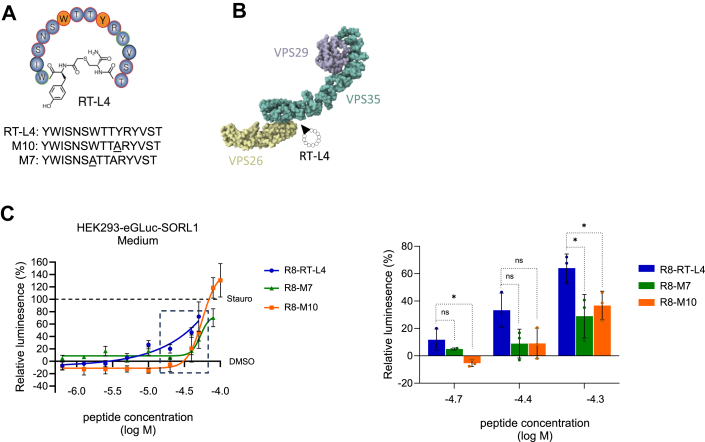
**Boosting retromer activity/recycling pathway using microcyclic peptides leads to increased eGLuc–SORL1 shedding.***A*, schematic diagram of RT-L4 microcyclic peptide, highlighting aromatic residues important for binding to retromer. Sequence alignment of RT-L4 and its variants M10 and M7 is shown, with substitutions underlined. *B*, relative binding position of RT-L4 cyclic peptide to retromer indicated on the structure of the mouse retromer complex (Protein Data Bank ID: 6VAC) (46). *C*, *left*, HEK293 cells stably expressing eGLuc–SORL1 (HEK293–eGLuc–SORL1) were treated with increasing concentrations of R8-conjugated RT-L4, or two mutated variants, R8-M10 and R8-M7 cyclic peptides, previously shown to have impaired ability to interact with retromer (35). Medium was harvested after 24 h, and the luminescence signal was measured. The *black dashed box* highlights the three highest nontoxic concentrations of R8–RT-L4 used for comparative analysis with the R8-M7 and R8-M10 peptides shown in the *right panel*. n = 1 independent experiments, with three technical replicates in each. Data are expressed as a percentage of luminescence signal in a double normalization over DMSO wells (0%) and staurosporine 50 nM wells (100%). Error bars indicate mean ± SD from experimental replicates within each condition. *Solid lines* represent fitted dose–response curves generated using a four-parameter logistic (variable slope) nonlinear regression model (“log[agonist] *versus* response – variable slope”) in GraphPad Prism. *Right*, comparison of R8–RT-L4–induced eGLuc–SORL1 shedding with the retromer-binding–deficient mutants R8-M7 and R8-M10 at the three highest nontoxic concentrations of R8–RT-L4. Data represent three independent experiments with three technical replicates each. Error bars indicate mean ± SD. Data were analyzed using a parametric two-tailed unpaired *t* test (∗*p* ≤ 0.05; ns, nonsignificant). DMSO, dimethyl sulfoxide; eGLuc, enhanced *Gaussia* luciferase; HEK293, human embryonic kidney 293 cell line; SORL1, sortilin-like receptor 1.

A recent study showed that the retromer complex can be stabilized using macrocyclic peptides (35). RT-L4 is one such peptide (Fig. 5*A*), which can bind to retromer with high affinity (*K*_*D*_ ∼80 nM) and stabilizes the complex assembly at the interface between the VPS26 and VPS35 subunits (Fig. 5*B*). Moreover, RT-L4 does not disrupt the association of retromer with its known binding partners (35). We hypothesized that enhancing the retromer activity through RT-L4–mediated complex stabilization would enhance the shedding of eGLuc–SORL1. To test this hypothesis, we generated a cell-penetrant derivative of the RT-L4 peptide by conjugation with the octaarginine (R8) tag (Fig. S4*A*), treated HEK293–eGLuc–SORL1 cells with R8-conjugated RT-L4 at noncytotoxic concentrations (Fig. S4*B*, see the *Experimental procedures* section), and measured the shedding of the reporter construct 24 h post-treatment. In three independent experiments, these studies indicated enhancement in the shedding of eGLuc–SORL1 in the conditioned media of cells treated with R8–RT-L4 at concentrations higher than 10^-5^ M, compared with the vehicle (DMSO)-treated controls (Figs. 5*C* and S4*C*). Although these data are promising, we encountered issues in the quantitative estimation of the cellular uptake of the cyclic peptides in HPLC analyses. The reason remains undetermined; however, one possible explanation is the sticky nature of the R8 tag and the hydrophobic peptide, which hinder these measurements. In parallel, we also tested the effects of two mutated versions of RT-L4, in which amino acids at position 7 or position 10 (M7 and M10, respectively, Fig. 5*A*) are replaced by an alanine and are reported to have impaired ability to stabilize the retromer complex (35). Treatment of HEK293–eGLuc–SORL1 cells with R8 derivatives of M7 and M10 led to less efficient boosting for the shedding of eGLuc–SORL1 compared with WT R8–RT-L4 at the three highest nontoxic concentrations (Figs. 5*C* and S4*C*; −4.7 log M, RT-L4 *versus* M7, *p* = 0.32 and RT-L4 *versus* M10, *p* = 0.021; −4.4 log M, RT-L4 *versus* M7, *p* = 0.061 and RT-L4 *versus* M10, *p* = 0.066; −4.3 log M, RT-L4 *versus* M7, *p* = 0.032 and RT-L4 *versus* M10, *p* = 0.032). This observed rank-order potency of the three cyclic peptides on the shedding of eGLuc–SORL1 reporter is suggestive of an effect through retromer stabilization.

**Figure 6:**
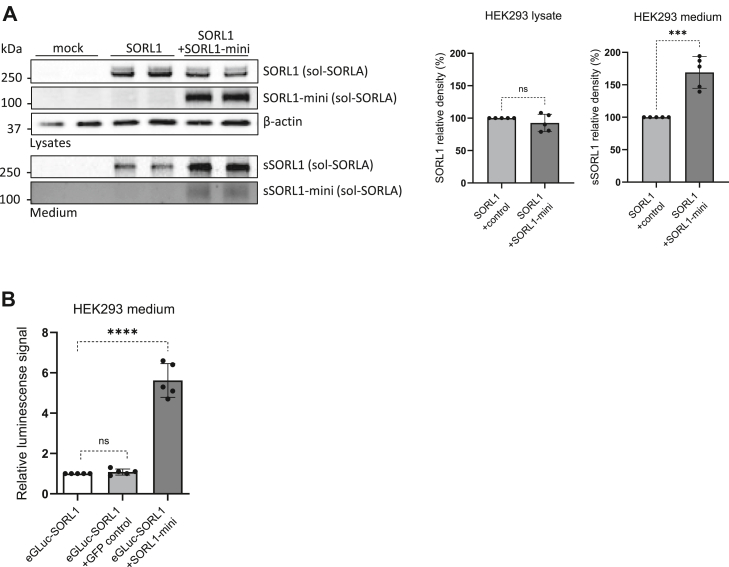
**Boosting retromer activity/recycling pathway using mini-SORL1 leads to increased eGLuc–SORL1 shedding.***A*, representative Western blot of cell lysate and conditioned media samples derived from HEK293 cells transfected with either SORL1 (along with an empty vector) or SORL1 plus minireceptor (SORL1-mini), detection antibodies as indicated. Corresponding densitometry analyses are shown; n = 5 independent experiments with two technical replicates in each. Data were analyzed using a parametric two-tailed paired *t* test (∗∗∗*p* ≤ 0.001). *B*, eGLuc activity measured in the media of HEK293 cells expressing either eGLuc–SORL1, eGLuc–SORL1 plus GFP control plasmid, or eGLuc–SORL1 plus minireceptor. n = 5 independent experiments with eight technical replicates in each. Error bars indicate mean ± SD. Data were analyzed using a parametric two-tailed unpaired *t* test (∗∗∗*p* ≤ 0.001; ns, nonsignificant). eGLuc, enhanced *Gaussia* luciferase; HEK293, human embryonic kidney 293 cell line; SORL1, sortilin-like receptor 1; sSORL1, soluble SORL1; sSORL1-mini, soluble minireceptor.

Previously, we have also demonstrated that endosome recycling activity can be enhanced by the expression of a truncated form of SORL1 containing the 3Fn-, transmembrane-, and cytoplasmic domains, termed the SORL1 minigene/minireceptor (SORL1-mini) (Fig. 1*A*) (7). This receptor fragment retains the property for forming homodimers as well as heterodimerizes with the full-length SORL1 protein and likely enhances the recycling activity by stabilizing the retromer complex at the endosome membrane (7). Hence, as a complementary approach, we wanted to assess whether SORL1-mini could increase recycling and thus shedding of the full-length SORL1. For this purpose, we transfected HEK293T cells to overexpress the native SORL1 with or without additional exogenous SORL1-mini. Western immunoblotting indicated that the coexpression of SORL1-mini significantly increased the presence of the shed sSORL1 fragment in the conditioned media (1.7-fold relative to controls; *p* = 0.0002) (Fig. 6*A*). In separate experiments, we performed cotransfection of eGLuc–SORL1 with minireceptor or with a GFP expression construct as a control and measured the luminescence signal in the conditioned media. Consistent with the shedding of untagged SORL1, we noted significant enhancement in eGLuc–SORL1 shedding by cells cotransfected with minireceptor, compared with the GFP control (4.6-fold relative to controls; *p* < 0.0001) (Fig. 6*B*).

**Figure 7:**
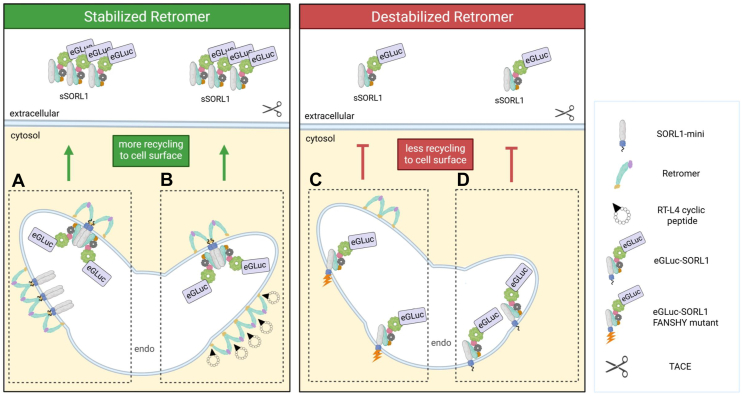
**eGLuc–SORL1 ectodomain shedding is dependent on retromer-mediated endosomal recycling.** Schematic comparing destabilized (*red panel*, *left*) *versus* stabilized (*green panel*, *right*) retromer conditions and their effects on eGLuc–SORL1 trafficking and ectodomain shedding. *A*, SORL1 minireceptor interacts and stabilizes retromer on the tubular membrane of the recycling endosomes, enhancing eGLuc–SORL1 dimerization, recycling to the plasma membrane and increasing ectodomain shedding. *B*, microcyclic peptide RT-L4 stabilizes retromer assembly on endosomal membranes and boosts eGLuc–SORL1 dimerization, recycling, and ectodomain shedding. *C*, mutation of the eGLuc–SORL1–FANSHY motif to six alanines (eGLuc–SORL1–FANSHY) disrupts its interaction with retromer, preventing efficient recycling of eGLuc–SORL1 from endosomes to the cell surface and reducing ectodomain shedding. *D*, siRNA-mediated knockdown of VPS35 depletes the retromer complex, which impairs eGLuc–SORL1 recycling to the plasma membrane, and decreases eGLuc–SORL1 ectodomain shedding. eGLuc, enhanced *Gaussia* luciferase; Endo, endosome, scissors indicating TACE (TNF-α-converting enzyme) cleavage; mini-SORL1, truncated SORL1 fragment containing 3Fn domains, transmembrane domain, and cytoplasmic tail; RT-L4, retromer-stabilizing microcyclic peptide; SORL1, sortilin-like receptor 1; TACE, tumor necrosis factor alpha–converting enzyme; TNFα, tumor necrosis factor alpha.

In conclusion, stabilizing retromer *via* R8–RT-L4 peptide treatment significantly enhances ectodomain shedding of the reporter. A similar increase was observed upon coexpression of SORL1-mini, suggesting that the minireceptor also stabilizes the retromer complex, although this remains to be experimentally validated. Conversely, decreasing retromer-dependent recycling through VPS35 siRNA knockdown or FANSHY>6A mutation markedly reduces eGLuc–SORL1 shedding (Fig. 7).

### Applying the eGLuc–SORL1 assay for variant pathogenicity

**Figure 8:**
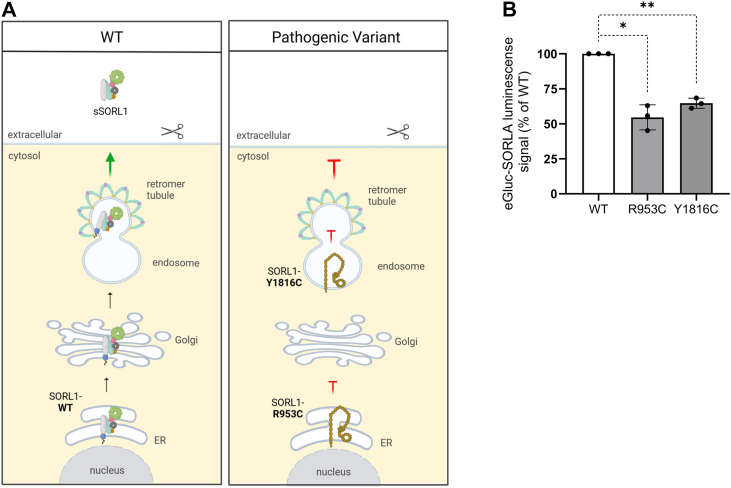
**eGLuc–SORL1 assay to assess genetic variant pathogenicity in Alzheimer’s disease.***A*, schematic comparison of the intracellular trafficking and shedding of WT and mutant eGLuc–SORL1 reporters. *Left* (*WT*), SORL1–WT is synthesized and folded in the endoplasmic reticulum (ER) and traffics through the Golgi to the endosomes. In endosomes, it interacts with the retromer complex and traffics *via* the recycling pathway to the cell surface. At the cell surface, tumor necrosis factor-alpha converting enzyme cleaves its extracellular domain, releasing soluble SORL1 (sSORL1) into the extracellular milieu. *Right (mutant)*, SORL1–R953C is misfolded and retained in the ER, preventing its delivery to endosomes. SORL1–Y1816C partially exits the ER and reaches endosomes but cannot engage with the retromer complex, blocking its recycling to the plasma membrane. Consequently, both mutants cannot traffic to the cell surface and will not be cleaved by TACE and will fail to release soluble sSORL1 into the extracellular milieu. *B*, eGLuc–SORL1 activity measured from conditioned medium of HEK293T cells expressing eGLuc–SORL1–WT or eGLuc–SORL1 with indicated pathogenic variants. Signal measured after 48 h of shedding. n = 3 independent experiments with two technical replicates in each. Error bars indicate mean ± SD. Data were analyzed using a parametric two-tailed paired *t* test (∗*p* ≤ 0.05; ∗∗*p* ≤ 0.01). eGLuc, enhanced *Gaussia* luciferase; Endo, endosome, scissors indicating tumor necrosis factor alpha–converting enzyme; HEK293T, human embryonic kidney 293T cell line; SORL1, sortilin-like receptor 1; TACE, tumor necrosis factor alpha–converting enzyme.

Recent reports suggest that pathogenic SORL1 variants linked to AD produce receptors with trafficking defects and reduced ectodomain shedding. For instance, p.R953C exhibits misfolding and abnormal retention in the ER, which prevents both trafficking to the endosomes and ectodomain shedding (18). A different SORL1 variant p.Y1816C partially exits the ER but fails to engage the retromer complex in endosomes (17). Pathogenic *SORL1* variants generally yield lower sSORL1 compared with WT SORL1 (19). This mechanistic model is illustrated in Figure 8*A*, depicting the deleterious impact of these two pathogenic variants on SORL1 trafficking and ectodomain shedding.

To demonstrate the utility of the eGLuc–SORL1 reporter assay for reliable quantification of the impact of genetic variants on ectodomain shedding, we introduced coding variant mutations for R953C and Y1816C in our reporter construct and compared their effects on shedding to the WT SORL1 reporter. Similar to our previous reports using Western blotting (17, 18), our reporter assay confirmed a significant reduction in shedding for both the genetic variants p.R953C and p.Y1816C (p.R953C: 44% reduction, *p* = 0.0127; p.Y1816C: 35% reduction, *p* = 0.0035) (Fig. 8*B*).

## Discussion

Here, we developed a method to reliably quantify the shedding of the endosomal sorting receptor SORL1, based on a fusion construct containing the stable eGLuc enzyme in cell-based assays. Subsequently, we used this new reporter system to provide evidence of how retromer activity is intimately linked to SORL1 shedding and showed how two independent methods (*i.e.*, cell-penetrant cyclic peptides and the SORL1 minigene) can be used to enhance endosome recycling as seen by elevated shedding of the reporter. In addition, the eGLuc–SORL1 reporter assay was successfully applied for assessing the decreased shedding of two dysfunctional *SORL1* mutants linked to AD, potentially implicating a future use of the assay for investigating the pathogenicity of novel genetic *SORL1* variants in the clinic.

The endosome recycling pathway has emerged as a major culprit for neurodegenerative disorders, including AD (36), and retromer plays essential functions to facilitate the export of the cargo proteins amyloid precursor protein and α-amino-3-hydroxy-5-methyl-4-isoxazolepropionic acid receptors out of the endosome (2, 9). We have recently demonstrated how retromer also plays a role in the maturation and processing of SORL1 (4, 22), which was unexpected based on how trafficking of SORL1 to the cell surface was previously assumed to follow the secretory pathway after its synthesis (20), a pathway where no function of the retromer core complex was identified/reported. Consistent with the aforementioned results, it is anticipated that a decrease in retromer would also lead to defects in SORL1 shedding. Indeed, a mouse model deficient in the retromer subunit VPS26 exhibits defects in SORL1 maturation (22), and a significant correlation between retromer levels and shed SORL1 in brains of patients with AD has also been recently reported (4). Here, using our novel reporter construct, we confirmed previous findings (22) that inhibition of endocytosis in cells with compromised retromer activity strongly impaired SORL1 shedding. Moreover, we demonstrate that boosting retromer-dependent endosomal trafficking—as exemplified by cyclic peptides with stabilizing effect on the retromer core complex—can oppositely increase shedding of SORL1. Combined, these findings suggest how shed sSORL1 can reflect intracellular retromer activity, and thus how our eGLuc–SORL1 reporter assay may be used for future searches for compounds that can modify retromer activity and thus the endosome recycling pathway, and perhaps serve as therapeutics for AD.

To date, quantification of sSORL1 has primarily been performed using Western immunoblotting, which is only semiquantitative, is time consuming, costly, and unsuitable for high-throughput applications. Here, we describe the development, validation, and application of a luminescent soluble SORL1 reporter assay that can be used as a surrogate indicator of the proteolytic processing of SORL1 through the retromer-dependent endosomal recycling pathway. The assay relies on the endogenous pathways of SORL1 processing, such that GLuc-tagged SORL1 ectodomain is shed into the extracellular milieu. Original GLuc catalyzes its luminescent reaction with a short half-life, leading to a relatively unstable and quickly decaying signal, making it unfavorable for high-throughput applications (27). However, in our reporter assay, we employed a variant of GLuc that demonstrates a slower decay of the GLuc luminescent signal (*i.e.*, eGLuc), which was achieved by mutating two oxidation-prone methionines (M43 and M110 (27, 28)). Furthermore, the eGLuc assay has a much reduced assay time (5–10 min *versus* hours or 1–2 days for Western blotting). Altogether, we expect this assay to be compatible with high-throughput applications, facilitating the discovery of compounds or genetic factors affecting the metabolism of WT SORL1.

Our assay may also be suitable for detecting changes in SORL1 ectodomain shedding induced by interacting adaptor proteins (APs) or ligands. While the retromer complex is a well-established mediator of SORL1 trafficking, several APs have been shown to interact with the SORL1 cytoplasmic tail either dependent on or independent of retromer, thereby influencing its intracellular localization and potentially its ectodomain shedding. For example, SNX27 binds to SORL1 and enhances its recycling to the cell surface, an effect that has been linked to reduced amyloidogenic processing (37), which may also impact shedding. Another recently identified AP is protein interacting with C-kinase 1 (PICK1), which interacts with the PDZ-binding motif at the C terminus of SORL1; although the physiological consequences of this interaction remain to be fully defined, it is suggested that PICK1 has a role in modulating SORL1’s trafficking itinerary (38). Additional APs, including the Golgi-localized, γ-ear–containing, ARF-binding (GGA) family (39, 40), phosphofurin acidic cluster sorting protein 1 (PACS1) (41), and AP complexes (20), have also been reported to bind to distinct motifs in the SORL1 cytoplasmic tail and contribute to its sorting within the endosomal–*trans*-Golgi network. Moreover, ligand binding has been shown to directly influence SORL1 shedding. For instance, the peptide ligand head activator was previously demonstrated to promote SORL1 ectodomain shedding in human neurons and neuroendocrine cells (42). We envision that the eGLuc–SORL1 reporter assay provides a sensitive platform to quantitatively assess such adaptor- or ligand-dependent effects. This highlights the broader applicability of the assay for identifying and characterizing novel regulators of SORL1 shedding in future studies.

In addition, we anticipate this assay to facilitate the discovery of *SORL1* pathogenic variants associated with AD. We have previously shown that *SORL1*-harboring pathogenic variants demonstrate reduced maturation and shedding of the receptor in the media of transfected cells (17, 18, 19). This is possibly caused by the retention of the misfolded protein in intracellular compartments of the early secretory pathway. sSORL1 is also present in the cerebrospinal fluid (CSF) and mainly originates from the shedding of neuronal SORL1 (43). Interestingly and consistent with the cell-based studies, carriers of pathogenic *SORL1* variants are expected to have decreased levels of sSORL1 in their CSF compared with carriers of nonpathogenic/common variants, as recently demonstrated for the p.Y1816C variant (17). Here, we suggest that measuring the level of sSORL1 production using the eGLuc–SORL1 assay is a rapid and effective way to further screen for *SORL1* variant pathogenicity, especially where CSF samples from variant carriers are unavailable.

While the assay execution is straightforward, a few notable limitations are worth considering. First, the assay is not a measure of cellular SORL1 expression but rather a surrogate indicator of soluble SORL1 ectodomain shedding, which can be affected by factors inherent to particular cell types under physiological or stress conditions (*e.g.*, TACE expression and/or activity). Second, the assay relies on the expression of a GLuc fusion construct, with the caveat that the efficiency of the expression may vary between cell lines. Hence, the results should be interpreted with caution and crossvalidated using ancillary measures (*e.g.*, LR11 and GLuc immunoblots—as we have performed—complemented with ELISA). In addition, to employ the reporter assay in more relevant biological contexts (*e.g.*, AD), it would be pertinent to validate its functionality using neuronal cells. Third, the overexpression of the luminescent reporter *per se* may affect the constitutive intracellular pathways of SORL1 processing; hence, it represents a confounding factor, for example, because of protein overload triggering ER stress response and/or impaired SORL1 trafficking to the membrane. Although in our cell culture studies we have established that the expression of SORL1 (WT or with GLuc) and ectodomain shedding is not significantly different (Fig. 1*B*), this may be a crucial factor for *SORL1* constructs containing mutations that may affect translational efficiency and/or half-life of the protein because of the biological consequences unique to specific *SORL1* variants.

## Experimental procedures

### Generation of eGLuc–*SORL1* construct

For generating the plasmid construct encoding SORL1 (WT) fused to the eGLuc, the two plasmids, pcDNA3 MYOC-eGLuc2 (44) and pcDNA3.1 *SORL1*–WT (21), were used as templates to amplify two insert DNA fragments (eGLuc2 and SORL1–WT) and a pcDNA3 backbone vector by PCR using Herculase II Fusion DNA Polymerase (Agilent Technologies, #600677). For this purpose, PCR primer pairs (Table 1) were used that contained 20 bp of additional sequence homologous to sequences flanking one of the ends of the three DNA fragments. Then the resulting DNA fragments were ligated by Gibson assembly (New England Biolabs, #E2621S) according to the manufacturer's instructions. The ligated end product (a pcDNA3 backbone vector containing eGLuc2 N-terminally attached to SORL1–WT; encompassing residues 82–2214 (29)) was transformed *into Escherichia coli DH5α*–competent cells. Then, a signal peptide was added to the N-terminal end of eGLuc2 (which was absent in the original pcDNA3 MYOC-eGLuc2 construct) using primers specified in Table 1. For this purpose, the signal peptide sequence (*Igκ leader*) was derived from pSecTag2B mammalian expression vector (a kind gift from Prof Peder Madsen [Aarhus University]) using MluI and SfiI restriction enzymes. Separately, MluI–SfiI restriction sites were introduced into the pcDNA3.1 eGLuc–SORL1 vector by PCR using unique primer pairs. After the MluI–SfiI restriction digestion of the two vectors, the *Igκ leader* and eGLuc2-SORL1–WT were gel-purified (0.5% agarose gel) using a commercial kit (Monarch DNA Gel Extraction Kit, #T1020S). The gel-purified products were then assembled using a ligation reaction (T4 DNA ligase; New England Biolabs, #M0202S). The ligated end product (a pcDNA3 backbone vector containing a signal peptide sequence and eGLuc N-terminally attached to SORL1–WT without its native signal peptide and propeptide [eGLuc–SORL1]) was transformed into *E. coli DH5α*–competent cells. The vector sequence was verified by Sanger sequencing.

**Table 1:** Primers used for generation of eGLuc–SORL1 and site-directed mutagenesis

Primers	Forward primer sequence (5′-3′)	Reverse primer sequence (5′-3′)
*SORL1* fragment	GATTACAAGGACGACAGCGCTGCCCTGCAGC	CCTGTACAGCTTGGAGGCTTTCAGGCTATCACCATGGGGAC
eGLuc fragment	CTCACCAAGCCTCTGCAAGCCCACCGAGAACAACGAA	CTGCAGGGCAGCGCTGTCGTCCTTGTAATCGTCACCAC
pcDNA3 vector	CCATGGTGATAGCCTGAAAGCCTCCAAGCTGTACAGG	GTTCTCGGTGGGCTTCAGAGGCTTGGTGAGGCTTC
Signal peptide (*Igκ leader*)	GCGGCCCAGCCGGCCAAGCCCACCGAGAACAACGAAG	AGTTATGTAACGCGGAACTCCATATATGG
Y1816C	GGCAATCTGACAGCTCATACATCCTGTGAGATTTCTGCCTGGGCCAAGACTG	CAGTCTTGGCCCAGGCAGAAATCTCACAGGATGTATGAGCTGTCAGATTGCC
R953C	GGATCACGTTCAGTGGCCAGCAGTGCTCTGTCATTCTGGACAACCTCC	GGAGGTTGTCCAGAATGACAGAGCACTGCTGGCCACTGAACGTGATCC

To generate the FANSHY>6A mutant vector, the pcDNA3.1 SORL1–FANSHY construct and pcDNA3.1 eGLuc–SORL1 were separately double digested with BstEII and XhoI, gel purified, ligated, and transformed *into E. coli DH5α*–competent cells as outlined above.

Introduction of genetic variants p.R953C and p.Y1816C was performed using a site-directed mutagenesis kit (Agilent Technologies, QuikChange II XL, #200522-5) according to the manufacturer’s instructions, with primers shown in Table 1.

All vector sequences containing respective mutations were verified by Sanger sequencing.

### Cell cultures and transient transfections

HEK293 and HEK293T cells were maintained in Dulbecco's modified Eagle's medium (4.5 g/L glucose; Gibco, #11965-084), CHO cells, both WT and with constitutional KO of alpha-1,3-mannosyl-glycoprotein 2-beta-*N*-acetylglucosaminyltransferase (*Mgat1 KO*; Henrik Clausen, University of Copenhagen, Copenhagen Center for Glycomics) were maintained in F-12 medium (Gibco, #11765054) and human neuroblastoma (SH-SY5Y) cells were maintained in Dulbecco's modified Eagle's medium/F-12 (Gibco, #11320033). All media were supplemented with 1% penicillin–streptomycin solution (Gibco, #15070063) and 10% fetal bovine serum (FBS). HEK293 cells stably expressing eGLuc–SORL1 were maintained in the following media: 42.5% MEM (ThermoFisher, #21090-022), 0.5% nonessential amino acid (ThermoFisher, #11140-035), 0.5% sodium pyruvate (ThermoFisher, #11360-039), 1% penicillin–streptomycin (ThermoFisher, #15140-022), 0.5% l-glutamine (ThermoFisher, 25030-024), 45% Ham's F12 (Sigma–Aldrich, #N6658-500ML), 10% heat-inactivated FBS (ThermoFisher, #10500-064), selection antibiotic puromycin (ThermoFisher, #A11138-2; used 1:10,000 in media final 1 μg/ml).

For transient transfections, approximately 500,000 cells/well were plated in a 6-well dish and allowed to attach overnight. The next day (20–24 h later), cells were transfected with eGLuc *SORL1* plasmid constructs (1.5 μg DNA/well) either using FuGene (Promega, #E2311) or Magnetofectamine O2 reagent (OZ Biosciences, #MTX2-0750), according to the manufacturer’s instructions. Subsequently (20–24 h later), the cell culture media were carefully removed and replaced with serum-free media (see above, for each cell line). Cell lysate and media samples were then collected for measuring luminescence signal (see below). Cells were washed once with ice-cold PBS (ThermoFisher, #10010031) and lysed in 100 μl of radioimmunoprecipitation assay buffer (25 mM Tris–HCl, pH 7.6; 150 mM sodium chloride) supplemented with NP-40 (1%), sodium deoxycholate (0.5%), sodium dodecyl sulfate (0.1%), and Triton-X (1%). Protein concentrations in the cell lysates were determined using bicinchoninic acid assay (ThermoFisher, #23225) according to the manufacturer’s instructions.

### Luminescence measurements

Samples (25–50 μl) containing conditioned media or aliquots of the cell lysates were transferred into a 96-well Nunc Microwell, flat-bottom, plate (ThermoFisher, #243656). Then, an equal volume of complete GLOW assay mix (NanoLight Technologies, #320), containing GLuc substrate coelenterazine prepared in NanoFuel GLOW buffer (see the manufacturer’s instructions) was added to each well, mixed with a multichannel pipette, spun the plate briefly, and incubated at room temperature for 5 min. Luminescence signal was acquired using either (a) CLARIOstar Plus multimode plate reader (BMG LABTECH) using with following parameters: shaking for 15 s at 600 rpm, integration time (4 s), read mode (top); or (b) EnVision PerkinElmer, luminescence with the following parameters: shaking for 15 s at 600 rpm (0.1 mm diameter orbital movement); aperture: ultrasensitive luminescence; distance between plate and detector: 0 mm; and measurement time: 0.1 s/well. Background signal was determined using conditioned media and cell lysates from nontransfected cells. Values were normalized to the protein input.

### Generation of stable cell line (HEK293–eGLuc–SORL1)

To generate HEK293 cells stably expressing eGLuc–SORL1, the PiggyBac transposon system was used. To this purpose, the eGLuc–*SORL1* coding sequence was cloned into a Mammalian Gene Expression PiggyBac Vector containing a murine stem cell virus promoter. This vector was then cotransfected with Super PiggyBac transposase vector (System Biosciences) into HEK293 cells, grown in a 6-well dish. Selection of cells expressing the reporter was performed using 1 μg/ml puromycin (ThermoFisher, #A11138-2).

### SDS-PAGE and Western blotting

Samples containing conditioned media (30 μl) or cell lysates (15 μg) were mixed with NuPAGE LDS sample buffer (Invitrogen, #2463558) supplemented with 80 mM DTT (ThermoFisher, #R0861). The samples were heated at 95 °C for 5 min and resolved on 4% to 12% acrylamide NuPAGE Bis–Tris gels (Invitrogen, #NP0322BOX). Proteins were transferred onto nitrocellulose membranes (Invitrogen, #IB23001) using the Invitrogen iBlot2 Gel Transfer System. The membranes were incubated (overnight, 4 °C) with primary antibody (mouse monoclonal anti-SORL1/LR11: BD Biosciences, #612633, 1:1000 dilution; Rabbit polyclonal GLuc: ThermoFisher, #PA1-181, 1:1000 dilution and Mouse monoclonal anti-β-actin: Sigma, #A5441, 1:5000 dilution). This was followed by incubation (1 h at room temperature) in swine anti-rabbit (Dako, #P0217, 1:2000 dilution) or goat anti-mouse (Dako, #P0260, 1:2000 dilution) horseradish peroxidase–conjugated secondary antibody. Detection was performed using the Pierce ECL kit (Thermo Scientific, #35055) and the Invitrogen iBright 1500 scanner. Quantification was performed using densitometry in ImageJ, National Institutes of Health (NIH) and data were plotted using GraphPad Prism (version 10.1.1).

### Automated Western blot with Jess system

VPS26b and VPS35 levels were measured in the lysate of HEK293 cells stably expressing eGLuc–SORL1 using an automated Western Jess system (Protein Simple, #004-650) according to the manufacturer’s instructions. Samples were loaded into a 12 to 230 kDa Jess Separation Module with 8 × 25 capillary cartridges (ProteinSimple, SM-W004). Primary antibodies used include Rabbit polyclonal anti-VPS26b (Novus, #NBP1-92575, 1:10 dilution) and Mouse monoclonal VPS35 (abcam, #ab57632, 1:100 dilution). Secondary antibodies used include Anti-Rabbit-horseradish peroxidase (Bio-Techne, #043-426, 1:20 dilution from 20× stock) for VPS26b detection and Anti-Mouse-horseradish peroxidase (Bio-Techne, #042-205, ready to use) for VPS35 detection. Final protein concentration of 0.25 μg/μl was used per well. Samples were incubated at 95 °C for 20 min. Instrument setting used: 40 min separation time, 90 min incubation of primary antibody, and 45 min incubation of secondary antibody. Total protein staining was used to normalize for protein loading (ThermoFisher, #TP-01).

### Dyn and PMA treatment

HEK293 cells stably expressing eGLuc–SORL1 were grown with a confluency of 530 cells/mm^2^ in a 384-well plate. The next day, the media were changed to start treatment with either 0.1% DMSO, Dyn monohydrate (Sigma, #D7693, 50 μM), and PMA (Sigma, #P8139, 160 nM). Dyn and PMA treatments were applied for 24 and 2 h, respectively, before refreshing the medium and subsequently measuring the luciferase activity. To determine whether compounds used in the study interfere directly with GLuc activity, N2a cells were transfected with the eGLuc–SORL1 reporter construct. After 24 h, conditioned media containing the shed reporter were collected and aliquoted. Two-fold (1:2) serial dilutions of PMA (60–0.5 μM), Dyn (80–2 μM), or scrambled siRNA (0.5–0.01 μM) were added directly to the media, and luminescence was measured after 30 min of incubation. No significant changes in eGLuc signal were observed compared with vehicle-treated controls, indicating that the compounds do not directly affect eGLuc enzymatic activity or light emission (Fig. S1).

### Minireceptor transfection

#### Full-length SORL1

HEK293 cells were plated at a density of 500,000 cells/well in a 6-well dish and allowed to attach overnight. The next day (20–24 h later), cells were transfected with the SORL1 full-length construct either with a control pcDNA construct (with no insert) or with the plasmid construct encoding the SORL1 minireceptor (as defined previously (7)) using FuGene HD (Promega, #E2311) transfection reagent according to the manufacturer’s instructions. Twenty-four hours later, the medium was changed to conditioned medium, and after 48 h, the cells and media were harvested and subjected to Western blotting.

#### eGLuc–SORL

HEK293 cells were plated in 96-well plates with a confluency of 1060 cells/mm^2^. After 24 h, cells were transfected with the eGLuc–SORL1 plasmid either alone, with a SORL1 minireceptor construct, or with a control GFP plasmid construct, using FuGene HD Transfection Reagent (#E2312). After 24 h, the media were collected, and luminescence was measured.

#### siRNA-mediated knockdown of VPS35

Predesigned siRNA was purchased for VPS35 (ThermoFisher, #AM16708, sense strand sequence: 5′-GCUCAACCUUGAACAUAUUtt-3′) and Cy3 Negative siRNA control (ThermoFisher, #AM4621), a nontargeting siRNA sequence with limited sequence similarity to known genes validated against human, rat and mouse genomes, and proven to have no significant effect on cell proliferation, viability, or morphology in cell-based screens. HEK293 cells stably expressing eGLuc–SORL1 at 70% to 80% confluency were transfected with control- or VPS35-siRNA using Lipofectamine RNAiMAX (ThermoFisher, #13778075) according to the manufacturer's instructions. Briefly, 12 nmol of siRNA was diluted in 100 μl of Opti-MEM and combined with 1 μl of Lipofectamine RNAiMAX, mixed gently, and transferred to each well of a 24-well plate and incubated at room temperature for 20 min. HEK293 cell suspension in a volume of 500 μl was then added to each well. After 72 h, the transfection was repeated. Another 72 h later, the media were changed to fresh media, and the cell lysate and medium were harvested for further analysis after 2 h.

### Immunocytochemistry and confocal microscopy

HEK293 cells were grown on poly-l-lysine-coated glass coverslips to reach a confluency of 50% to 60%. After 24 h, cells were transfected with 1.5 μg of plasmid DNA encoding either SORL1 or eGLuc–SORL1 per well using FuGene according to the manufacturer’s instructions. After another 24 h, cells were fixed with 4% paraformaldehyde, followed by two washes with PBS containing 0.1% Triton-X and blocked in blocking buffer (PBS [pH 7.4], FBS 10%) for 30 min at room temperature. Next, cells were incubated overnight at 4 °C in a primary antibody against SORL1: either rabbit polyclonal anti-SORL1 (pAb_5387; Aarhus University, 1:300 dilution) or mouse monoclonal anti-SORL1 (mAb_AG4; Aarhus University, 1:100 dilution) along with one of the following antibodies: anti-EEA1 (BD Biosciences, #610457, 1:200 dilution), anti-VPS35 (Everest Biotech, EB06268, 1:200 dilution), anti-GM130 (BD Transduction Laboratories, #51-9001978, 1:300 dilution), or anticalnexin antibody (abcam, #ab22595, 1:300 dilution). The next day, coverslips with cells were washed in PBS containing 0.1% Triton-X and incubated in Alexa-Fluor secondary antibodies (Invitrogen, 1:500 dilution) for 1 h at room temperature. Coverslips were mounted on glass slides using mounting medium containing 4′,6-diamidino-2-phenylindole (Sigma, #DUO82040) for staining of nuclei. All images were obtained using a Zeiss LSM800 confocal microscope and processed using Zen 3.5 (ZEN lite) software. Colocalization was quantified using the JACOP plugin in ImageJ software, with results presented as Mander’s correlation coefficient. Graphing and statistical analysis were performed with GraphPad Prism 10.1.1.

### R8-conjugated RT-L4 cyclic peptide treatment

HEK293 cells stably expressing eGLuc–SORL1 were seeded in 96-well plates with an approximate confluency of 3000 cells/mm^2^ and treated with 0.1% DMSO, 50 nM staurosporine, or with increasing concentrations (0.6–250 μM) of R8-conjugated RT-L4 cyclic peptide (custom made by Wuxi) and harvested 24 h after treatment. Staurosporine was included as a positive control to establish a reproducible upper bound (100%) reference signal for double normalization of shedding. At this low, nontoxic concentration, staurosporine does not induce apoptosis but consistently enhances shedding through a mechanism that remains to be elucidated. This robust and reproducible effect makes it a valuable internal control for defining the dynamic range of shedding responses across experiments. The media of the cells were then subjected to a luciferase assay. Data points with treatment concentrations above 50 μM were excluded because of toxicity concerns based on qualitative evaluation of brightfield and DRAQ7/Hoechst 33342 imaging as well as quantitative assessment of cytotoxicity (Fig. S4).

### Statistics

The data were statistically analyzed in GraphPad Prism software (version 10.1.1) and presented as the mean ± SD. Data were analyzed by paired or unpaired Student's *t* test, as indicated in the respective figure legends. Statistical significance was reached with a *p* value of less than 0.05, indicated as *p* ≤ 0.05 (∗), *p* ≤ 0.01 (∗∗), *p* ≤ 0.001 (∗∗∗), *p* ≤ 0.0001 (∗∗∗∗), or deemed not significantly (ns) changed.

## Data availability

All data are included in the article and supporting information. Additional information is available from the corresponding author upon reasonable request.

## Supporting information

This article contains supporting information (35).

## Conflict of interest

The authors declare that they have no conflicts of interest with the contents of this article.
