# Supplementary material for: A luminescent reporter assay to quantify SORL1 ectodomain shedding and retromer-dependent endosome recycling activity
Source: J Biol Chem. 2026 Jan 8;302(2):111136. doi: 10.1016/j.jbc.2026.111136 (PMC12860930; doi:10.1016/j.jbc.2026.111136)
Supplement: Supporting Information [file mmc1.pdf]

Supporting Information Fig. S1

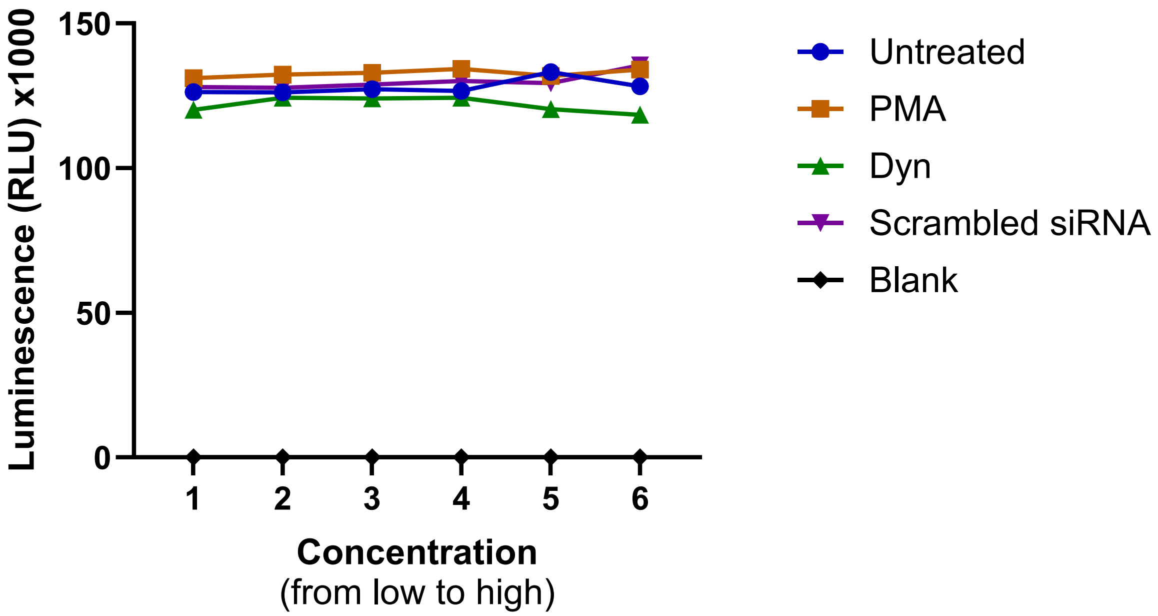

Supporting Information Figure S1. Gaussia luciferase activity measured in conditioned medium from N2a cells expressing eGLuc-SORL1 following direct addition of two-fold serial dilutions Phorbol 12-myristate 13-acetate (PMA; 60 to 0.5 µM), Dynasore (Dyn; 80 to 2 µM), or scrambled siRNA (0.5 to 0.01 µM). Untreated samples (blue) and blank medium without eGLuc (black) were included as controls. Compounds were added directly to aliquoted conditioned medium for 30 min prior to luminescence measurement. n = 1 independent experiment with two technical replicates per condition.

Supporting Information Fig. S2

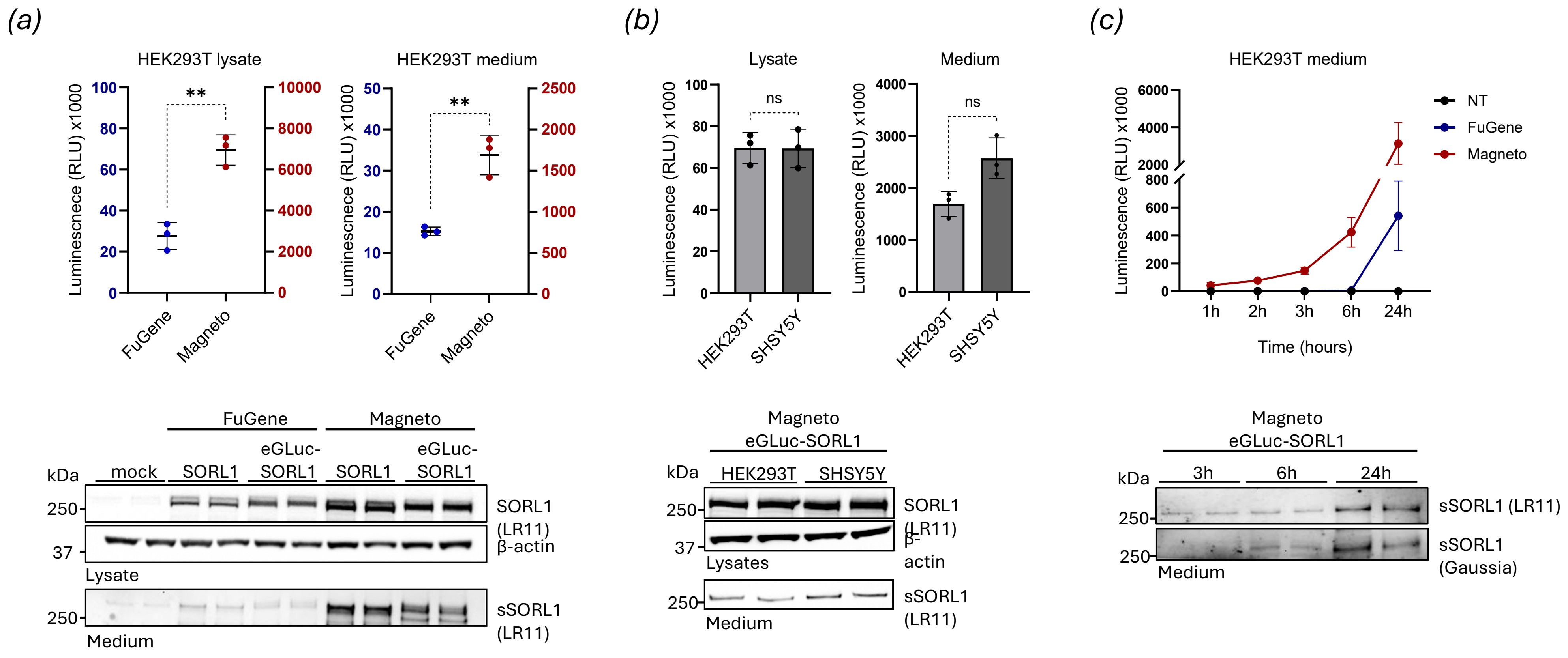

Supporting Information Figure S2. Different methods of eGLuc-SORL1 delivery and eGLuc assay conditions. ***(a)*** eGLuc activity measured from cell lysates and conditioned medium from HEK293T cells expressing eGLuc-SORL1 following transfection with FuGene or Magnetofectamine, measured after 24 h of shedding. n=3 independent experiments with two technical replicates in each. Error bars indicate mean±SD. Data were analyzed using parametric two-tailed paired t test ($p \leq 0.01$; **). Representative immunoblots of cell lysates and conditioned medium from HEK293T cells expressing SORL1 and eGLuc-SORL1 following transfection using antibodies as indicated. sSORL1; soluble SORL1. ***(b)*** eGLuc activity measured in the cell lysate and conditioned medium of HEK293T FuGene or Magnetofectamine reagents. Detection and SH-SY5Y cells expressing eGLuc-SORL1 following transfection with Magnetofectamine reagent, measured after 24 h of shedding. n=3 independent experiments with two technical replicates in each. Data were analyzed using parametric two-tailed paired t test. (ns; not significant). Representative immunoblots of cell lysates and conditioned medium from HEK293T and SH-SY5Y cells expressing eGLuc-SORL1 following transfection with Magnetofectamine reagents. Detection antibodies as indicated. ***(c)*** eGLuc activity measured at 1, 2, 3, 6 and 24 hours in the conditioned media of HEK293T cells expressing eGLuc-SORL1 following transfection with FuGene or Magnetofectamine reagents. n=1 independent experiments with two technical replicates in each; (NT; non-transfected)**.** Representative immunoblots of conditioned media from HEK293T cells following transfection with eGLuc-SORL1 using Magnetofectamine reagents. Detection antibodies as indicated.

Supporting Information Fig. S3

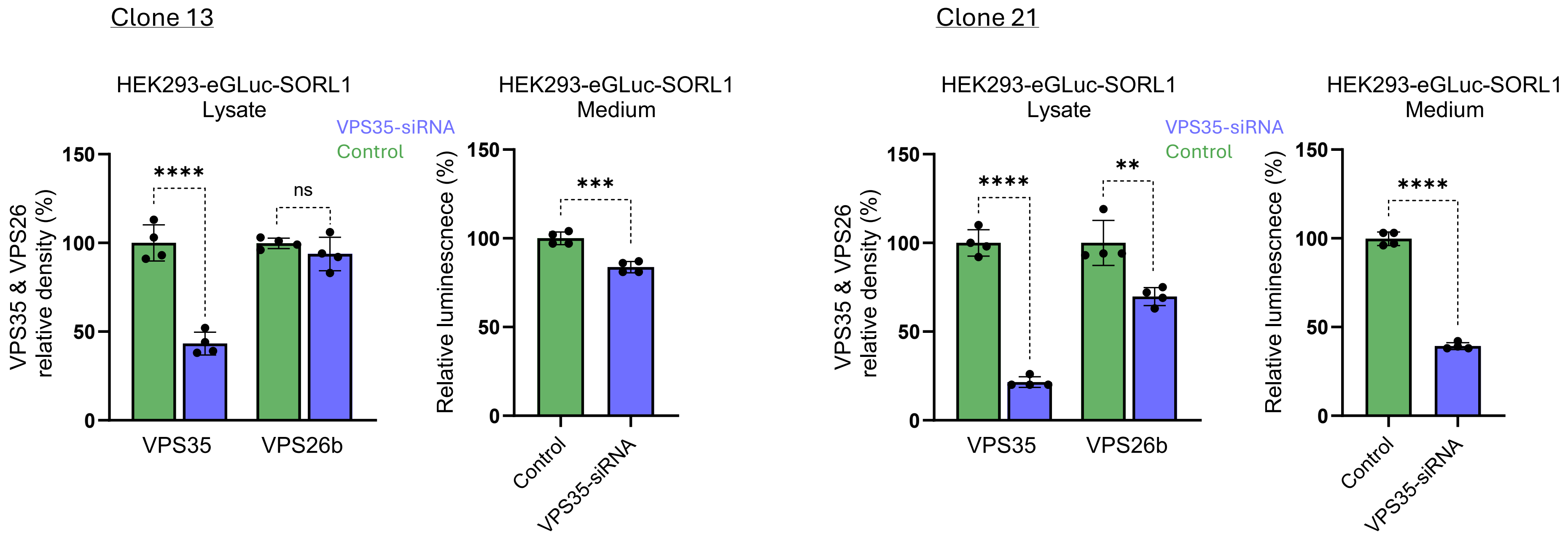

Supporting Information Figure S3. Capillary Western densitometry of VPS35 and VPS26b in lysates from HEK293 cells stably expressing eGLuc-SORL1 (HEK293-eGLuc-SORL1; Clones 13 and 21), and eGLuc activity measured in conditioned medium from the same cells, following treatment with VPS35 siRNA (blue) or non-targeting/scrambled siRNA (green). n=1 independent experiments with four technical replicates in each. Error bars indicate mean±SD. Data were analyzed using parametric two-tailed unpaired t test and shown as relative to the scrambled control (ns; not significant; p ≤0.01; **, p ≤0.001; ***, p ≤0.0001; ****).

Supporting Information Fig. S4

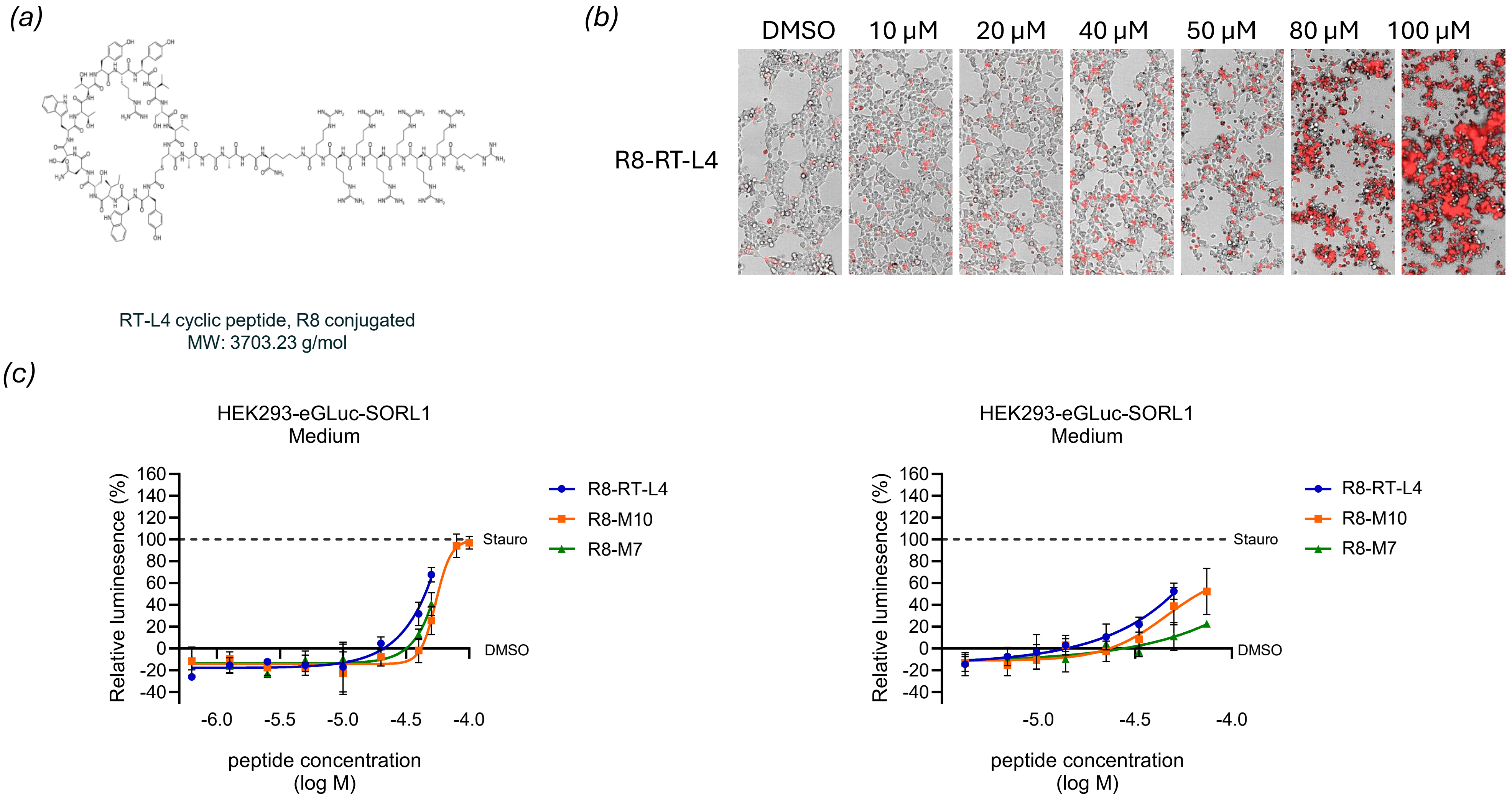

Supporting Information Figure S4 ***(a)*** Chemical structure of the RT-L4 cyclic peptide conjugated with an octa-arginine (R8) tag. ***(b)*** Representative microscopic images of HEK293 cells stably expressing eGLuc-SORL1 treated with increasing concentrations of R8-conjugated RT-L4 cyclic peptide for 24 h. After removing the media cells were stained with staining solution (Hoechst33342 1:2000, DRAQ7 1:200) for 15 minutes at 37°C and imaged directly using Operetta system. ***(c)*** Biological replicates of HEK293 cells stably expressing eGLuc-SORL1 (HEK293-eGLuc-SORL1) treated with increasing concentrations of R8-conjugated RT-L4 or two mutated variants, R8-M10 and R8-M7 cyclic peptides, which have been shown to have impaired ability to interact with Retromer (35) Medium was harvested after 24 h and luminescence signal was measured. n=1 independent experiments, with three technical replicates in each. Data are expressed as percentage of luminescence signal in a double normalization over DMSO wells (0%) and Staurosporine 50 nM wells (100%). Error bars indicate mean±SD from experimental replicates within each condition. Solid lines represent fitted dose–response curves generated using a four-parameter logistic (variable slope) nonlinear regression model ("log(agonist) vs. response – variable slope") in GraphPad Prism.
